# Pauci- and Multibacillary Leprosy: Two Distinct, Genetically Neglected Diseases

**DOI:** 10.1371/journal.pntd.0004345

**Published:** 2016-05-24

**Authors:** Jean Gaschignard, Audrey Virginia Grant, Nguyen Van Thuc, Marianna Orlova, Aurélie Cobat, Nguyen Thu Huong, Nguyen Ngoc Ba, Vu Hong Thai, Laurent Abel, Erwin Schurr, Alexandre Alcaïs

**Affiliations:** 1 Laboratory of Human Genetics of Infectious Diseases, Necker Branch, INSERM U1163, Necker Hospital for Sick Children, INSERM, Paris, France, EU; 2 Paris Descartes University, Imagine Institute, Paris, France, EU; 3 Unité de Génétique fonctionnelle des maladies infectieuses, Institut Pasteur, Paris, France, EU; 4 Hospital for Dermato-Venerology, Ho Chi Minh City, Vietnam; 5 Program in Infectious Diseases and Immunity in Global Health, The Research Institute of the McGill University Health Centre, Montreal, Quebec, Canada; 6 The McGill International TB Centre, Departments of Human Genetics and Medicine, McGill University, Montreal, Quebec, Canada; 7 URC, CIC, Necker and Cochin Hospitals, Paris, France, EU; Fondation Raoul Follereau, FRANCE

## Abstract

After sustained exposure to *Mycobacterium leprae*, only a subset of exposed individuals develops clinical leprosy. Moreover, leprosy patients show a wide spectrum of clinical manifestations that extend from the paucibacillary (PB) to the multibacillary (MB) form of the disease. This “polarization” of leprosy has long been a major focus of investigation for immunologists because of the different immune response in these two forms. But while leprosy per se has been shown to be under tight human genetic control, few epidemiological or genetic studies have focused on leprosy subtypes.

Using PubMed, we collected available data in English on the epidemiology of leprosy polarization and the possible role of human genetics in its pathophysiology until September 2015. At the genetic level, we assembled a list of 28 genes from the literature that are associated with leprosy subtypes or implicated in the polarization process. Our bibliographical search revealed that improved study designs are needed to identify genes associated with leprosy polarization. Future investigations should not be restricted to a subanalysis of leprosy per se studies but should instead contrast MB to PB individuals. We show the latter approach to be the most powerful design for the identification of genetic polarization determinants. Finally, we bring to light the important resource represented by the nine-banded armadillo model, a unique animal model for leprosy.

## Introduction

Leprosy is a chronic infectious disease caused by *Mycobacterium leprae*, with over 200,000 new cases detected each year, often leading to severe disabilities and social stigma. After sustained exposure to *M*. *leprae*, only a subset of individuals develops clinical leprosy. From the early observations of familial aggregation of leprosy cases to the most recent genome-wide association studies identifying genetic polymorphisms associated with leprosy, there is strong evidence that the development of the disease is under tight human genetic control [1,2]. The interest in the genetics of leprosy is reflected by the continuous increase in the number of genetic publications (host and pathogen), while publications on immunology of leprosy have decreased significantly after 1980 [3].

There is considerable clinical variability among leprosy patients, with a clinical spectrum that extends from the polar “tuberculoid” to the polar “lepromatous” form of the disease [4,5]. The tuberculoid form is characterized by a small number of hypopigmented, well-bordered, anesthetic skin lesions with a low bacillary load (therefore this form is commonly referred to as “paucibacillary” [PB]), early peripheral nerve impairment, and a T-helper 1 (Th1)–mediated immune response. In contrast, the lepromatous form is characterized by numerous infiltrated skin lesions displaying high bacillary loads (therefore referred to as “multibacillary” [MB]), impaired peripheral nerves, possible involvement of internal organs, and a Th2-mediated immune response [6]. Leprosy has long been a major focus of investigation for immunologists because it is an excellent example *in natura* of the clinical impact of a differential immune response of B and T lymphocytes on the form of disease, but it has not been thoroughly considered by geneticists. Some genetic studies have focused specifically on either PB leprosy or MB leprosy; however, very few have investigated the process that we call “polarization,” which is the process that drives the differentiation to different subtypes and therefore impacts on all or at least multiple subtypes of leprosy.

From a genetic perspective, polarization can thus be regarded as a neglected phenotype. To address this shortcoming, we have conducted a review of epidemiological studies addressing leprosy subtypes and polarization. We argue that the poor understanding of the genetic architecture of leprosy polarization is mainly caused by inappropriate study designs. We provide suggestions for future research on PB and MB leprosy and propose that the use of the armadillo model will be of pivotal importance for the study of these phenotypes.

## Methodology

### Review of the literature

On September 30th, 2015, we searched PubMed with the search terms “(leprosy OR *Mycobacterium leprae* OR multibacillary OR paucibacillary OR lepromatous OR tuberculoid NOT tuberculosis) AND (genetic OR association OR risk factors)” and identified ~1,500 publications on leprosy (~1,000 on genetics and ~500 on epidemiology). Epidemiological data were also gathered from the Weekly Epidemiological Records released by the World Health Organization (WHO).

## Results and Discussion

### Leprosy classification

As early as 600 b.c., the *Sushruta-Samhita* (the most ancient Indian medical book), distinguished *kushta*, a lepromatous form of the disease, from *rakta*, a neural form [7]. In 1881, Danielssen and Boeck distinguished a nodular and an anesthetic leprosy [8], and in 1895, Hansen and Looft used the terms “anesthetic” and “maculo-anesthetic” [9]. In 1935, Wade noticed that some patients could move from a tuberculoid state to a lepromatous one, and named “borderline” the form of leprosy of these patients [10]. Nowadays, once the diagnosis of leprosy is made, the identification of the clinical form is critical because it will determine the length of multidrug therapy [11]. In addition, the clinical determination of leprosy subtype provides major pathophysiological information, which was explored early on by immunologists but, as already mentioned, was somewhat disregarded by the genetics community.

International Leprosy Conferences in Cairo (1938), La Havana (1948), and Madrid (1953) paved the way for the subclassification of the disease into three classes, based on clinical (aspect of lesions), bacteriological (identification of bacilli), immunological (lepromin test), and histological (nature of the infiltrate, grouping of bacilli) criteria, known as the Madrid definition. The three Madrid classes are: Tuberculoid (T), Borderline (or Dimorphic) (B), and Lepromatous (or Virchowian) (L). The Madrid classification scheme is still used in some countries like Brazil [12]. A major development came with the leprosy classification by Ridley and Jopling in 1966, which aimed to better characterize the borderline group that could comprise patients with different clinical, immunological, bacteriological, or histological findings. Ridley and Jopling proposed five subtypes: Tuberculoid Tuberculoid (TT), Borderline Tuberculoid (BT), Borderline Borderline (BB), Borderline Lepromatous (BL), and Lepromatous Lepromatous (LL) [13]. The latter form may itself be refined in “subpolar” (LLs) and “polar” (LLp) on a clinicopathologic basis [14].

To facilitate diagnosis and treatment of leprosy in the field, WHO proposed in 1982 a simplified classification. It relied on clinical findings and the bacteriological index (BI), calculated by counting 6 to 8 stained smears under the 100x oil immersion lens in a smear made by nicking the skin with a sharp scalpel and scraping it. The results are expressed on a logarithmic scale from 0 to 6+ [15]. The WHO-82 classification distinguishes PB leprosy, which includes TT and BT patients with BI ≤ 1, from MB leprosy, which comprises BB, BL, and LL patients, as well as those with a BI ≥ 2 [11]. In 1988, WHO changed its bacteriological criteria and any patient with a positive BI was now classified as MB (“WHO-88” definition) [16]. Finally, in 1996, WHO switched for an operational definition to optimize treatment allocation: patients with less than five skin lesions are diagnosed as PB, while all others are diagnosed as MB (“WHO-96” definition). A side effect is that this operationally driven classification has eclipsed pathophysiological aspects differentiating the leprosy forms. A summary of all classifications is given in Box 1.

**Box 1. Different Classifications of Leprosy**

**Madrid (1953):** T (Tuberculoid), B (Borderline) and L (Lepromatous), according to clinical (skin and nerve lesions), bacteriological (bacteriological index [BI]—a measure of the number of acid-fast bacilli in the dermis expressed in a logarithmic scale), immunological (lepromin reaction), and histological (infiltrate’s nature) criteria.

**Ridley–Jopling (1966):** TT (Tuberculoid Tuberculoid), BT (Borderline Tuberculoid), BB (Borderline Borderline), BL (Borderline Lepromatous), and LL (Lepromatous Lepromatous), defined according to clinical, bacteriological, immunological, and histological criteria.

**WHO (1982):** Paucibacillary (PB) or Multibacillary (MB) according to Ridley–Jopling (or Madrid) classifications and the BI. PB = TT + BT (or T) and BI ≤ 1; MB = BB + BL + LL (or B + L) or BI ≥ 2.

**WHO (1988):** PB = TT + BT and BI = 0; MB = BB + BL + LL or BI ≥ 1.

**WHO (1996):** PB or MB according to the sole number of skin lesions (≤/>5).

*****

**Polarization:** process that drives an infected individual towards the PB or the MB form (in our manuscript, irrespective of the classification used to define PB and MB cases).

**Fig 1:**
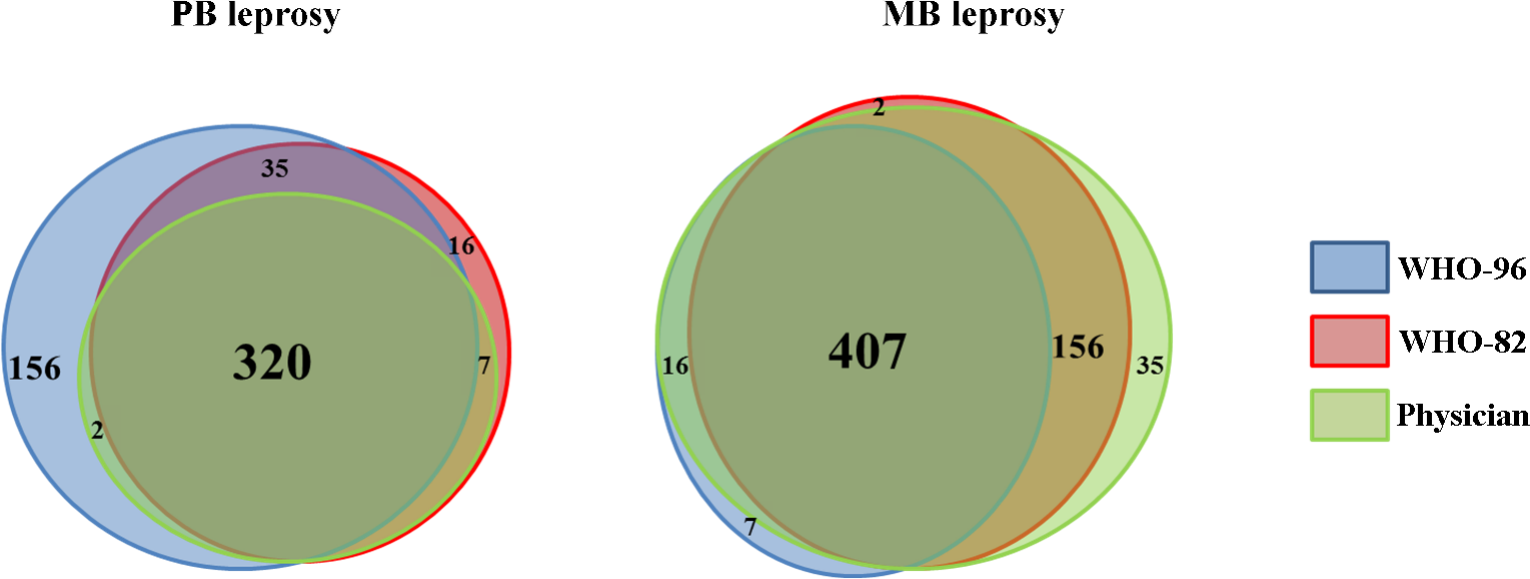
Concordance of leprosy polarization phenotypes according to WHO-82, WHO-96, or physician definitions in a Vietnamese sample. Venn diagram for the number of patients with PB leprosy (left) and MB leprosy (right) according to three definitions: WHO-82, WHO-96, and physician in a Vietnamese sample. The surfaces of overlap are approximately proportional to the number of individuals identically classified by one, two, or three definitions. There was no individual classified PB by a physician and MB by WHO-82 + WHO-96 as well as no individual classified MB by a physician and PB by WHO-82 + WHO-96.

To which extent the evolution of the WHO classification system impacted on the counts of PB and MB forms is unclear. In a Vietnamese sample of 1,182 leprosy cases, 1,127, 1,074, and 1,051 could be classified according to WHO-82 (mainly relying on Ridley and Jopling criteria), WHO-96 (which only relies on the number of lesions), and the physicians, respectively (S1 Appendix). The physician classification was the one that in practice determined which multidrug regimen each patient received. The proportion of MB patients was 66% (695/1,051), 60% (672/1,127), and 41% (435/1,074) with the final physicians’, WHO-82, and WH0-96 classifications, respectively. Fig 1 shows the Venn diagram of MB and PB patients classified according to the three definitions. The concordance analysis of the three classifications was quantified by means of the Kappa coefficient for the subset of 943 individuals classified for all three definitions: it was higher between “WHO-82” and “physician” than between “WHO-82” and “WHO-96” or between “WHO-96” and “physician” (0.88 [0.85–0.91], 0.62 [0.58–0.67], and 0.59 [0.54–0.64], respectively—a common interpretation of the Kappa coefficient is: poor [0–0.4], medium [0.4–0.6], good [0.6–0.8], and excellent [0.8–1]).

By defining MB and PB forms, an implicit phenotype is the contrast between these two forms. We define this implicit phenotype as leprosy polarization, i.e., the process that drives the development of leprosy subtypes, which needs to be differentiated from factors that impact on only one subtype. Here, we will refer to polarization whenever PB and MB forms are opposed, irrespective of the criteria used to define PB and MB leprosy.

## Epidemiology of Leprosy Subtypes

No epidemiological study specifically dealing with MB or PB leprosy has been published. Therefore, we based our approach on more general epidemiological studies of leprosy per se that included information on MB and PB forms. The vast majority of recent epidemiological studies focused on the contacts of an index leprosy case to identify risk factors for the occurrence of secondary cases [17–21]. In this setting, subgroup analysis of clinical forms is not very powerful, given the low number of such secondary cases: indeed, the detection rate of new cases is often less than 1% after 4 years of follow-up, requiring 10,000 contacts to ultimately study 100 secondary leprosy cases [18,19]. Since there is minimal variability within the genome of *M*. *leprae*, [22–24], pathogen factors are unlikely to contribute to clinical variability. Therefore, we restricted our explorations to host-related risk factors: sex, age, geographical origin, and bacille Calmette-Guérin (BCG) vaccination status.

### Sex

The number of leprosy cases reported in adults is consistently higher among men, with a male to female sex ratio ranging between 1.5 and 2 [25]. Few exceptions of a sex ratio <1 have been described in some African countries such as Burkina Faso, Kenya, or Uganda [26]. Exposure, reporting, or access to care biases cannot fully explain such a difference: during a massive outbreak on the island of Nauru in the Pacific (where leprosy affected a third of the population during the 1920s and where these biases are expected to play a minor role), the sex ratio was still 1.5 [27]. Concerning the sex ratio for MB or PB forms of the disease, a very large Brazilian study that used census data from the Ministry of Health between 2006 and 2010 (40,544 PB and 29,764 MB cases) showed a sex ratio larger than 2 for MB patients but slightly lower than 1 for PB cases (Madrid classification) [28]. This might explain why, according to the relative proportion of MB and PB forms in a population, the overall sex ratio varies widely and why in some African countries, where the PB form is predominant, women are overrepresented among leprosy cases.

### Age

**Fig 2:**
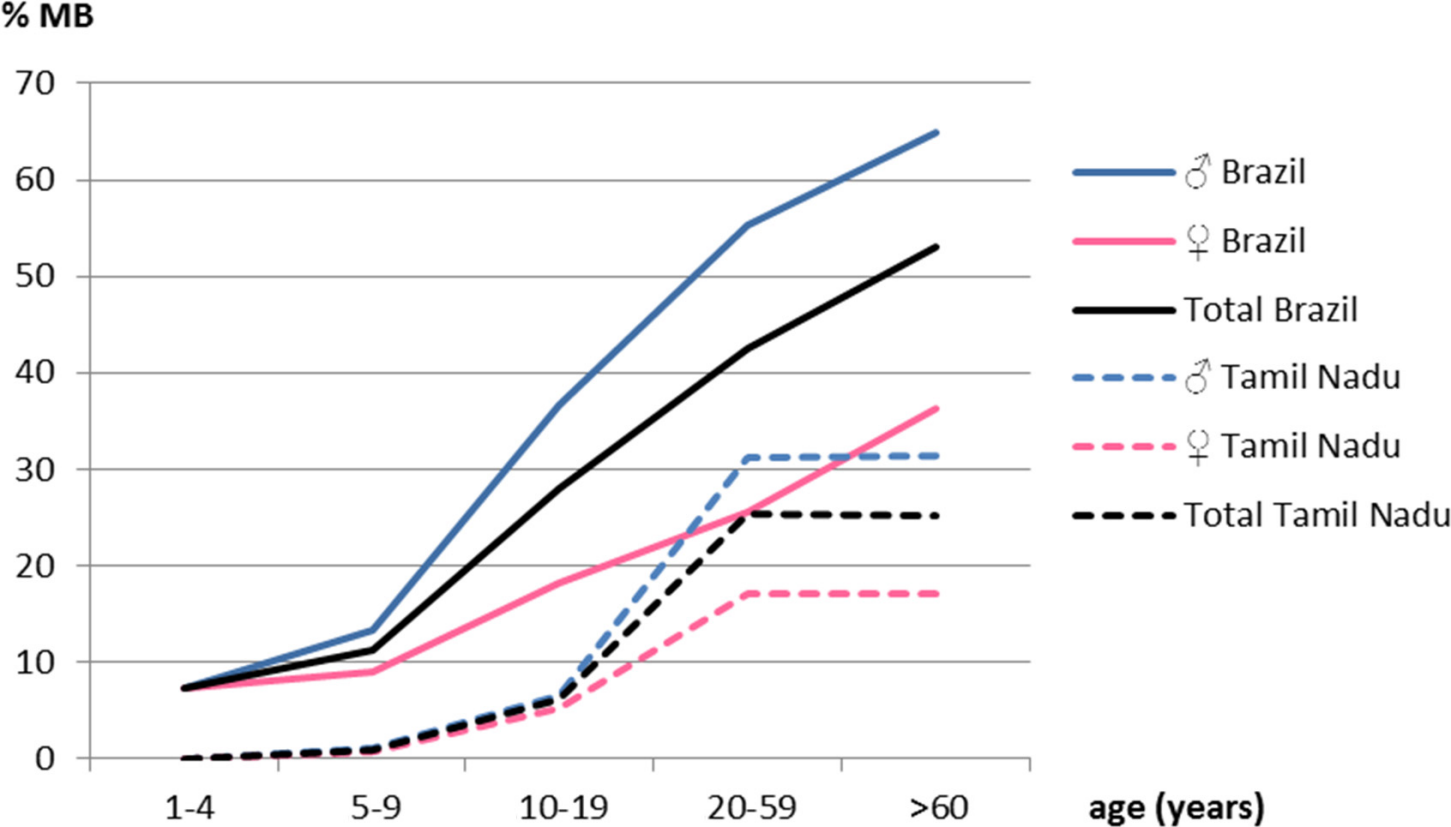
Proportion of multibacillary patients according to age and sex. Proportion of multibacillary patients according to age and sex are given for two distinct geographic areas: (1) in Brazil, 40,544 PB and 29,764 MB cases (Madrid classification) were declared over the 2006–2010 period (national data) [28]; (2) in Tamil Nadu (India), during the 1962–1970 period, 3,963 PB and 1,258 MB cases were collected out of a total of 276,568 persons screened for leprosy (Madrid classification) [31].

Generally, the mean age of MB cases is higher than the one of PB cases, the common explanation being one of a longer period of incubation for the MB form (reviewed in [29]). A study in Andhra Pradesh (India) in 1962 showed that the proportion of MB cases—corresponding to L/(L+T) with the Madrid classification—increased from less than 5% between 5 and 9 years of age to over 25% for patients 25–64 years of age [30]. These proportions were similar to those observed during the same period in another region from India (Tamil Nadu) [31] and in Burma (both using the Madrid definition) (Fig 2) [28]. An independent study conducted in Uttar Pradesh (India) between 2005 and 2010 found the proportion of MB cases to be 54% in children against 77% in adults (*p* < 0.001) [32]. Finally, we used the data of the Brazilian study mentioned above [28] to show that the proportion of MB leprosy continuously increased with age, from less than 10% before 10 years of age to more than 50% for patients older than 60 years (Fig 2) [28]. The influence of age on the proportion of MB patients was greater in men than in women, and multivariate analysis showed that the effects of these two factors (age and sex) on leprosy polarization were independent (Fig 2). In a Vietnamese sample previously mentioned, we also observed that age was correlated with a higher proportion of MB patients, and this correlation was stronger for males than for females (S1 Fig).

### BCG

Two meta-analyses have shown that BCG vaccination significantly reduced the risk of developing clinical leprosy [33,34]. They included 26 and 28 studies, respectively, that used various classifications of MB and PB leprosy (Madrid, Ridley–Jopling, WHO-82, WHO-88, and WHO-96); 22 studies were common to both meta-analyses. The proportion of the observed protection ranged from 26% to 61%, depending on the studies included. In the first meta-analysis, the protective effect was greater for MB than for PB patients (78% versus 59%, *p* = 0.04). In contrast, in the second study, in which an adjustment was made on the number of immunizations with BCG and on the type of study (observational versus interventional), the impact of BCG was not significantly different on the onset of one leprosy form or the other. A few reports suggested that the LL form is insensitive to vaccination (as these patients are not able to develop a granulomatous response) [35], while others suggested that BCG vaccination may lead exposed individuals to preferentially develop a PB rather than a MB leprosy [36,37].

### Geography

The proportion of MB cases varies greatly across continents but also within each continent, between neighboring countries: among the countries that reported more than 1,000 cases in 2012, the proportion of MB cases (WHO-96) varied between 45% (Bangladesh) and 92% (Philippines) [25]. It is unlikely that demographic data such as age and sex explain the observed variability, given that population sex ratios are similar across countries, and the patient age distribution is relatively homogeneous within a continent.

### Endemicity

In 1966, Kenneth Newell, an epidemiologist self-defined as “naïve” about leprosy, published a seminal paper entitled “An Epidemiologist's View of Leprosy.” He focused on a largely disregarded observation that he considered to be very informative: in countries of high endemicity, the prevalence of lepromatous cases (similar to MB cases as defined by Madrid classification) was relatively stable (between 5 and 10/1,000). This observation enabled him to disregard some debated hypotheses about leprosy pathogenesis: (1) the lepromatous form is caused by a more virulent strain of *M*. *leprae*, (2) lepromatous cases are associated with a greater inoculum and/or greater exposure, and (3) polarization is linked to an environmental factor common to all human populations. Conversely, the data were consistent with the hypothesis that a fixed proportion of the infected population presents one or several characteristics that make certain persons more likely to develop a lepromatous form of leprosy. It is therefore reasonable to consider host genetic factors as a plausible explanation of leprosy polarization [38].

## Human Genetics of Leprosy Subtypes

There are many methodological tools in genetic epidemiology that can be used according to the hypotheses to be tested and the nature of the data. Their main characteristics are summarized in S1 Table [39]. A number of approaches do not require any biological material and rely only on the information and the knowledge of the familial relationships. These DNA-independent studies are of particular interest in the context of neglected tropical diseases and knowledge transfer to endemic countries: they are easier to implement and intrinsically technology-independent (i.e., their cost has remained low). The following section illustrates how such DNA-independent methods have contributed to our current understanding of the genetics of leprosy subtypes.

### DNA-independent analyses: familial correlations, twin and segregation studies

Numerous studies have established that among close contacts of index cases (e.g., individuals living in the same house or sharing the same bed as the case), those biologically related to the index case are significantly more at risk of developing leprosy per se [17,18,21]. Intriguingly, no study has focused specifically on the subtype of leprosy developed by these secondary cases according to their demographics (age, sex, etc.,) or those of the index case, further substantiating that these phenotypes are neglected. Conversely, twin studies considered subtypes in addition to leprosy per se [40,41]. Among them, a study of twin pairs in which at least one twin suffered from the MB subtype of leprosy (Madrid classification) found a higher concordance rate of the MB subtype in monozygotic (MZ) as compared to dizygotic (DZ) pairs (70% in MZ versus 20% in DZ) [40]. On the opposite, for the PB form, concordance rates between MZ and DZ twins were very close. As we mentioned earlier, there is a greater proportion of MB leprosy among males [25], and this probably marginally increased the concordance rate in MZ twins versus DZ ones (which may be of opposite sex). Finally, several familial segregation studies in the Philippines, La Desirade Island, Thailand, and Brazil support a significant host genetic contribution to both MB and PB forms of the disease, although the genetic models were not always concordant across studies [41–44].

### DNA-based analyses: linkage and association studies

#### Candidate-region or genome-wide linkage studies

Early linkage studies using DNA were performed on small chromosomal segments around a particular gene of interest chosen for its known function (“candidate-gene,” or more precisely, “candidate-region” hypothesis). While the vast majority of these studies focused on leprosy per se, one analyzed the genetic control of leprosy polarization as defined by Ridley and Jopling [45]. It included 118 sibs (i.e., brothers and/or sisters) from 20 Vietnamese families and covered 6 chromosomal regions of interest: 1p36 around tumor necrosis factor receptor 2 (*TNFR2*), 5q31–q33 around a group of cytokines, 6p21 around *TNF*, 12p13 around *TNFR1*, 12q13 around vitamin D receptor (*VDR*), and 10p13. Polarization was coded as a “quantitative” trait, giving an increasing weight throughout the Ridley–Jopling spectrum (TT<BT<BB<BL<LL). The study detected significant linkage of the 6p21 region, including *TNF*, with leprosy polarization [45].

Studies from Jamieson and Miller in a Brazilian sample reported linkage of leprosy per se with chromosomal regions 17q11–q21 [46], 6p21, 17q22, and 20p13 [47]. These studies subsequently defined two groups, T* and L*. The T* group included patients classified as TT, BT, and BB according to the Ridley–Jopling scale, while the L* group comprised BL and LL patients. Using this uncommon classification (BB usually comes along with BL and LL in the MB group), significant linkage of the 17q11 and 20p13 chromosomal regions was detected for the L* phenotype but not for the T* group. Finally, a linkage study from Malawi found a significant linkage signal between polarization and genetic markers on the 21q22 [48] chromosomal region (WHO-88 definition). However, as for most regions identified by linkage, the functional importance of specific genes located in this region awaits confirmation.

The first genome-wide (i.e., no restriction to a particular genomic region) linkage study of leprosy was performed in India in 2001. The sample included 224 families comprising 245 sib pairs affected by leprosy [49]. A significant linkage hit (*p* < 2·10^−5^) for leprosy was observed with genetic markers located on chromosomal region 10p13. However, because of the very high proportion of PB cases (98%; Madrid definition), it was not possible to decide whether the mapped locus influenced leprosy per se or was specific for the PB form. A strong argument for the impact of the 10p13 region on leprosy subtypes rather than leprosy per se is the significant heterogeneity found in a Vietnamese sample between PB and MB individuals (WHO-88, *p* = 0.03) [50]. A second study in India added some families to the sample of the first linkage study (243 families in total, including 233 with only PB children) and identified a second linkage signal on the 20p12 chromosomal region [51]. Overall, the results of the linkage studies in Indian and Vietnamese leprosy patients suggest that the 10p13 and 20p12 regions are differentially implicated according to the subtype considered.

#### Candidate-gene, candidate-region, and genome-wide association studies

Numerous studies have tested the association between leprosy per se and genetic variants located in biologically relevant candidate genes. These studies were mainly focused on genes from the major histocompatibility complex (MHC) region, the Toll-like receptors (TLRs), and the cytokine family [1,2]. Similarly to linkage studies, only a subset of the candidate-gene studies has considered the clinical form of the disease. Their results are summarized in Table 1.

**Table 1:** Studies identifying 28 genes associated with clinical forms of leprosy or its polarization.

Comparison of MB against PB individuals (*)						
**Gene**	**Country**	**Phenotype**	**Definition**	**Number of individuals(MB/PB/Controls)**	***p***	**Marker (allele, genetic model)**
*MBL2* [57]	Nepal	MB versus PB	WHO-88	581/343/101	0.01	polymorphism G161A (recessive)
*LTA4H* [66]	Ethiopia	MB versus PB	WHO-88	443/335/0	0.001	rs1978331 T/C and rs2660898 T/G
***LACC1*** [81]	**China**	**MB versus PB**	**WHO-88**	**305/397/1,225**	**<0.00001**	**rs3764147 and rs10507522 (additive)**
***LRRK2*** [81]	**China**	**MB versus PB**	**WHO-88**	**305/397/1,225**	**0.0008**	**rs1491938 (additive)**
***NOD2*** [81]	**China**	**MB versus PB**	**WHO-88**	**305/397/1,225**	**0.00004**	**rs9302752 (additive)**
***RIPK2*** [81]	**China**	**MB versus PB**	**WHO-88**	**305/397/1,225**	**0.0008**	**rs42490 (additive)**
*TLR4* [65]	Ethiopia	MB versus PB	WHO-88	298/138/197	0.05	polymorphism T1196C (C allele, dominant)
*NRAMP1* [75]	Mali	MB versus PB	WHO-88	181/92/201	0.003	3′ UTR 4-base pair [bp] insertion/deletion TGTG (het versus del/del)
*MBL2* [56]	Brazil	MB versus PB	Madrid	150/36/214	0.01	
*IL-10* [61]	Brazil	MB versus PB	WHO-96	166/131/283	0.006	Haplotype -375A/-2849G/-2763C
*VDR* [67]	India	MB versus PB	Madrid	124/107/165	0.001	TaqI T -> C
*IL-10* [62]	Brazil	MB versus PB	WHO-96	143/59/62	<0.01	Haplotype -375A/-2849G/-2763C
*MICA* [60]	Brazil	MB versus PB	Madrid	153/45/201	0.01	MICA*010 and MICA*027
*IL-12RB2* [104]	Japan	MB versus PB	Madrid	130/46/68	<0.01	A-1035G (G allele, allelic)A-1023G (G allele, allelic)-650delG (G allele, allelic)A-464G (G allele, allelic)Haplotype -1035A/-1023A/-650G/-464A
*TLR2* [53]	Malawi	MB versus PB	WHO-96	26/184/379	0.04	microsatellite 224pb (in intron 2) (undefined model)
*IL-17F* [79]	India	MB versus PB	WHO-88	88/52/84	<0.05	T7488C (TT versus TC)
*IL-10* [105]	Brazil	MB versus PB	Madrid	65(LL)/43(TT)/240	<0.01	haplotype -1082G/-819C/-592C
*LAMA2* [76]	Indonesia	MB versus PB	WHO-88	27/26/58	<0.005	T7809C or V2587A (TC versus TT)
*KIR2DS3* [69]	Brazil	LL versus TT	Ridley-Jopling	65(LL)/42(TT)/289	0.04	
Comparison of one leprosy subtype (MB or PB) individuals against controls (*)						
**Gene**	**Country**	**Phenotype**	**Definition**	**Number of individuals(MB/PB/Controls)**	***p***	**Marker (allele, genetic model)**
*IL-23R* [77]	India	PB versus Controls (CTL)	WHO-88	416/427/1,502	0.02	Copy Number Variant (CNV)—77 base amplicon located at exon 11 of the gene
*MRC1*[58]	Brazil	MB versus CTL	WHO-88	373/331/396	0.001	rs1926736 (G/A = G396S, G allele, recessive)rs2437257 (C/G = F407L, C allele, recessive)haplotype G396-F407
*CCDC122* [83]	Vietnam	MB versus CTL	WHO-88	286/188	0.006	rs3088362 (C/A, A allele, additive)
*HLA-DR-DQ* [83]	Vietnam	MB versus CTL	WHO-88	286/188	0.0003	rs602875 (A/G, A allele, additive)
*LACC1* [83]	Vietnam	MB versus CTL	WHO-88	286/188	0.0007	rs3764147 (A/G, G allele, additive)rs10507522 (A/G, A allele, additive)
*TLR2* [52]	Ethiopia	MB versus CTL	WHO-88	298/138/187	0.02	microsatellite 288bp (dominant)
*TNF* [54]	India	MB versus CTL	Madrid	121/107/160	0.01	G308A (A allele, allelic)
*RIPK2* [84]	India	PB versus CTL	WHO-96	137/74/230	0.02	haplotype GA for rs40457 and rs42490
*LRRK2* [84]	India	PB versus CTL	WHO-88	137/74/230	0.00001	rs1873613A/G (A allele, allelic)
*VDR* [68]	India	MB vs CTL	WHO-96	135/87/182	0.001	rs7975232 (= ApaI, genotypic)TaqI-FokI-ApaI (haplotype T-F-a and T-F-A)
*VDR* [68]	India	PB vs CTL	WHO-96	135/87/182	0.01	TaqI-FokI-ApaI (haplotype T-f-a)
*LTA* [53]	Malawi	MB versus CTL	WHO-96	26/184/379	0.003	5′ UTR microsatellite (AC/GT)n at -3.5kilobase (101-bp allele)
*HLA-G* [64]	Brazil	MB versus CTL	WHO-88	76/70/128	0.02	polymorphism + A3187G (A allele, dominant)
*MICA* [59]	China	MB versus CTL	Undefined	50/19/112	<0.05	MICA-A5 (allelic)
*IFNγ* [63]	Brazil	PB versus CTL	Madrid	59/10/98	0.01	CA repeat in intron 1 (<122bp = "short," 122–126bp = "long") at risk = "long"
*DEFB1* [77]	Mexico	MB versus CTL	Madrid	46/18/151	0.02	G668C (C allele, dominant)
*TNF* [55]	Thailand	MB versus CTL	WHO-96	24/13/140	0.04	G308A (A allele, undefined model)
*OPA1* [78]	China	LL versus CTL	Ridley-Jopling	109(LL)/175(TT)/583	0.002	rs414237 (A allele, genotypic)rs9838374 (C allele, genotypic)
*C4A* [72]	Thailand	LL versus CTL	Ridley-Jopling	71(LL)/27(TT)/201	<0.01	F1 allele ("functionally inactive")
*HSPA1A* [70]	India	TT versus CTL	Ridley-Jopling	0/49(TT)/38	0.03	allele at risk HSP70-1A (allelic)

* The table is divided into two panels: upper panel groups are studies that analyzed the polarization phenotype and lower panel groups are those that compared MB or PB patients to controls. Studies are sorted according to their decreased sample size. Bold lines correspond to genes that were identified by GWAS, and underlining corresponds to studies whose main outcome was to identify genes specific to leprosy polarization or a polar form of leprosy. Hence, all black lines correspond to studies designed to study leprosy per se and a subsequent subgroup analysis for MB, PB, or other polarization phenotypes.

The strategy of association studies with a candidate gene faces the classical problem of “multiple testing” (several genes tested and several variants within each gene), which can result in inflated type I errors if not properly corrected. To avoid inflated type I errors in genetic association studies, replication (i.e., confirmation in an independent sample from the same population) and validation studies (confirmation in a sample from a different population) are critical. The problem of inflated type I errors in candidate gene association studies is not specific to leprosy but extends to the whole field of complex traits. The only validated studies for leprosy subtype (whether PB, MB, or polarization) are provided by single nucleotide polymorphisms (SNPs) in genes for Toll-like receptor 2 (*TLR2*) in Ethiopia [52] and Malawi [53], *TNF* in India [54] and Thailand [55], mannose-binding lectin 2 (*MBL*2) in Brazil [56] and Nepal [57], mannose receptor C type 1 (*MRC1*) in Brazil and Vietnam [58], and MHC class I chain-related gene A (*MICA)* in China [59] and Brazil [60]. Finally, the association of interleukin 10 (*IL-10*) with polarization has been replicated in two independent Brazilian samples [61,62].

Other associations have only been found once, and the implication of the tested genes in leprosy pathogenesis must be considered with caution: *IFNγ* and *HLA-G* in Brazil [63,64]; *TLR4* and leukotriene A4 hydrolase (*LTA4H*) in Ethiopia [65,66]; vitamin D receptor (*VDR)* [67,68], killer cell immunoglobulin like receptor, two Ig domains and short cytoplasmic tail 3 (*KIR2DS3*) [69], heat shock protein 1A (*HSPA1A*), and *IL-23R* in India [70,71]; lymphotoxin-α (*LTA*) in Malawi [53]; complement fraction 4 (*C4A*) in Thailand [72]; *IL-12B* in Mexico [73]; *IL-12RB2* in Japan [74]; natural resistance-associated macrophage protein 1 (*NRAMP1*) in Mali [75]; laminin 2 (*LAMA2*) in Indonesia [76]; defensin-β1 (*DEFB1*) in Mexico [77]; and optic atrophy 1 (*OPA1*) in China [78]. Interestingly, for VDR, one study identified a polymorphism associated with leprosy when comparing MB to PB [67], while a second study identified a haplotype when comparing MB to controls and another haplotype when comparing PB to controls [68]. Finally, a Brazilian study illustrated the impact of clinical classification. Here, the association of genetic variants in the gene coding for IL-17F was significant for leprosy polarization based on the WHO-88 classification (88 PB and 52 MB patients, odds-ratio [OR] 3.2, *p* < 0.05) but not for the WHO-96 definition (53 PB and 87 MB individuals) [79]. As mentioned above, linkage of the 10p13 chromosomal region with the PB form of leprosy in India [49] has been confirmed in Vietnam [50]. To characterize the molecular identity of the genes responsible for the signal in the region, a fine-mapping association study of this candidate region was conducted using 1,522 markers and spanned over nine megabases (Mb), excluding *MRC1* [80]. The primary sample included 294 nuclear families from Southern Vietnam with 303 sibs affected by leprosy, of which 63% were classified as MB (WHO-96 definition). The replication sample from the same area consisted of 192 trios (2 parents and 1 child with leprosy), with 55% MB children. Three SNPs located in the genes coding for nebulette (*NEBL*, actin-binding Z-disk protein) and cubilin (*CUBN*, intrinsic factor-cobalamin receptor), located 3 Mb apart, were specifically and independently associated with the MB form [80].

The first genome-wide association study (GWAS) for leprosy was published in 2009 [81]. It included 706 cases and 1,225 controls from China; 4,367 additional controls were added in a subsequent study published in 2011 [82]. 500,000 SNPs spanned over the entire genome were analyzed in this sample. The most promising signals were tested in a replication sample including 3,254 cases and 5,955 controls, mainly from Eastern China but also from Chinese ethnic minorities. In total, significant association of leprosy was detected with 18 polymorphisms assigned to eight genes: *HLA-DR-DQ*, receptor-interacting protein kinase 2 (*RIPK2*), tumor necrosis factor superfamily member 15 (*TNFSF15*), coiled-coil domain containing 122 (*CCDC122*), LACcase containing domain 1 (*LACC1*), nucleotide-binding oligomerization domain containing 2 (*NOD2*), Rab32 protein (*RAB32*), and *IL-23R* [81,82]. Heterogeneity in strength of association between the MB and PB groups (WHO-88) was observed for four polymorphisms assigned to four genes (*LACC1*, *LRRK2*, *NOD2*, and *RIPK2*). Validation studies of the observed associations were conducted in Vietnamese and Indian samples. In a familial sample from Vietnam including 286 MB and 188 PB leprosy patients (WHO-88), signals located in *HLA-DR-DQ*, *RIPK2*, *CCDC122*, *LACC1*, and *NOD2* were validated (*p* < 0.05), and the effect was stronger in MB patients for *HLA-DR-DQ*, *CCDC122*, and *LACC1* [83]. In the Indian case-control study using a smaller population (137 MB and 74 PB cases—WHO-88), signals in *LRRK2* and *RIPK2* genes were validated, and the observed effect was consistently more significant for PB patients [84]. A second study using 48,000 markers located in 2,092 genes was performed in an Indian population in 2010. The primary sample included only 258 cases and 300 controls from North India. Markers in the *TLR1* and *HLA-DRB1/DQA1* genes were significantly associated with leprosy per se. The test for heterogeneity between MB and PB groups (WHO-88) was not significant for the association with markers of these two genes [85]. Nonetheless, in both GWAS, leprosy per se was the main phenotype, and only SNPs that were significant with this phenotype were subsequently tested for heterogeneity between MB and PB groups. This is not an optimal design (see next section) and may well have missed SNPs with significant impact on polarization.

## Study Design: Benefits of Optimal Hypothesis Testing

**Fig 3:**
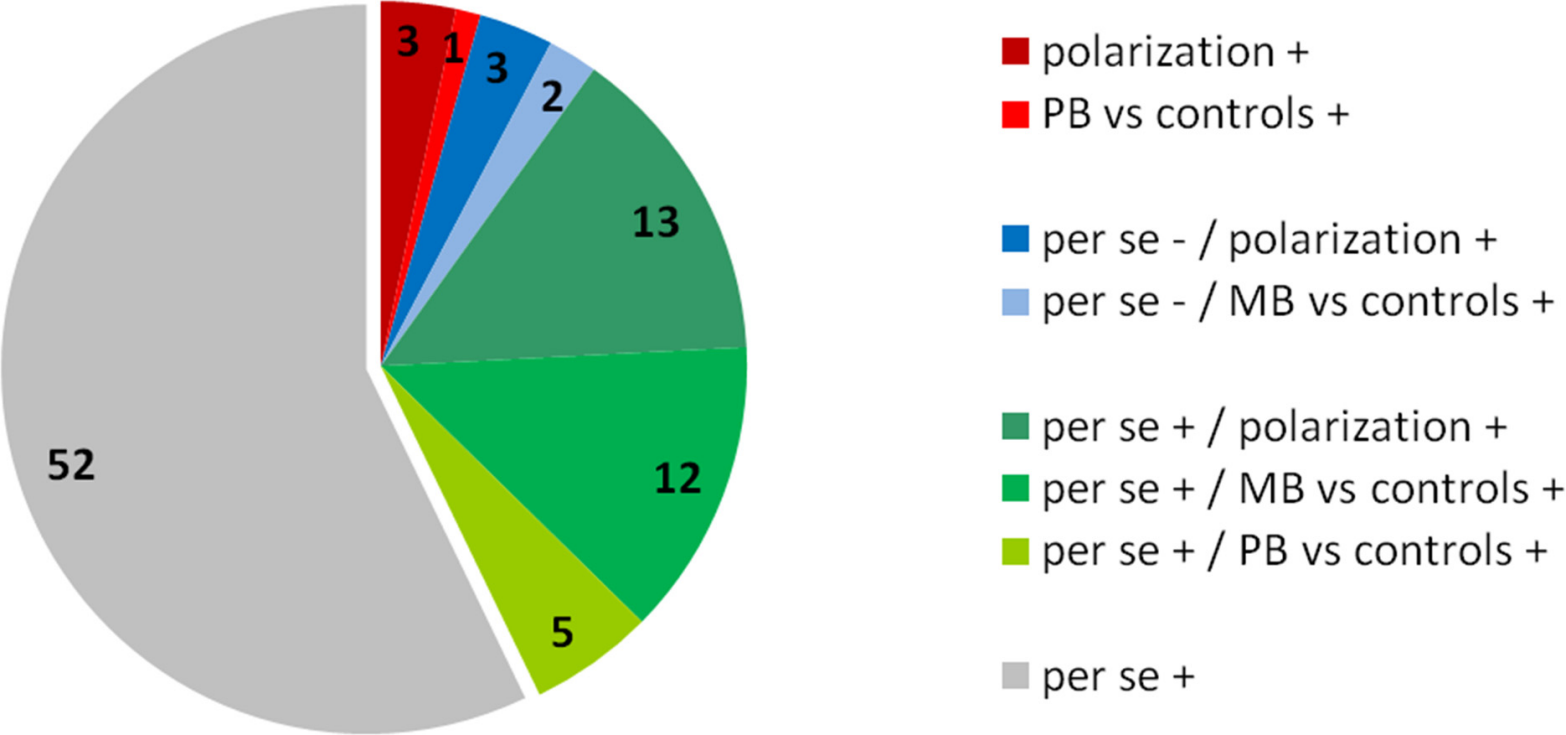
Distribution of 91 published associations between a genetic variant and leprosy per se, any leprosy subtype (i.e., MB or PB), or polarization. The + and–symbols refer to the presence or the absence of association of a given gene with one of the leprosy subtypes in the study. The red quadrant refers to studies for which the primary outcome was the association with leprosy polarization or the PB subtypes; the blue quadrant refers to studies that could not conclude an association with leprosy per se but did conclude an association with one of its subtypes; the green quadrant refers to studies that identified an association for both leprosy per se and one of its subtypes; and the grey quadrant refers to studies for which an association with only leprosy per se was reported.

The number of genes for which a variant has been associated in one of leprosy’s subtypes or its polarization (39 associations in 28 genes) (Table 1) is shorter than for leprosy per se (82 associations in 50 genes). Fig 3 summarizes the distribution of the associations according to leprosy phenotypes. Several scenarios may explain the difference. First, since most studies were conducted in samples used for leprosy per se studies, by design patient numbers for subtype analysis were smaller than for leprosy per se, resulting in lower power to detect genetic variants impacting on leprosy subtype. This is consistent with the fact that most reported associations concerned the MB form, which is more common in endemic countries except India. Second, an inadequate definition of MB and PB forms can also lead to a loss of power, as illustrated by the study of *IL-17F* [79]. Third, most studies analyzing leprosy polarization did not account for demographic risk factors: yet, the study of leprosy per se has revealed that the inclusion of these covariates was essential (e.g., the association of leprosy per se with *PARK2* or *LTA* was three to five times stronger in young patients [86,87]).

Schematically, most studies first look for association by comparing leprosy cases to controls, then derive a *p*-value for a given genetic marker and a given genetic model (*p*_per se_). Only in a second step are additional tests for significance of association by comparing MB to all controls (*p*_MB_) and PB to all controls (*p*_PB_) conducted. If *p*_MB_ < *p*_per se_ < *p*_PB_, one might conclude that this SNP is associated with leprosy and has a major effect on MB patients (if *p*_MB_ < *p*_per se_ < threshold) or that this SNP is associated with MB leprosy only (if *p*_MB_ < threshold < *p*_per se_). Such a general strategy has several major shortcomings, including the well-known fact that *p*-values contain little meaningful information: odds ratios should be compared instead. Second, correction for multiple testing should be performed when testing for leprosy subtypes after testing for leprosy per se. Third, heterogeneity tests (used to compare PB versus MB) are generally not very powerful.

**Fig 4:**
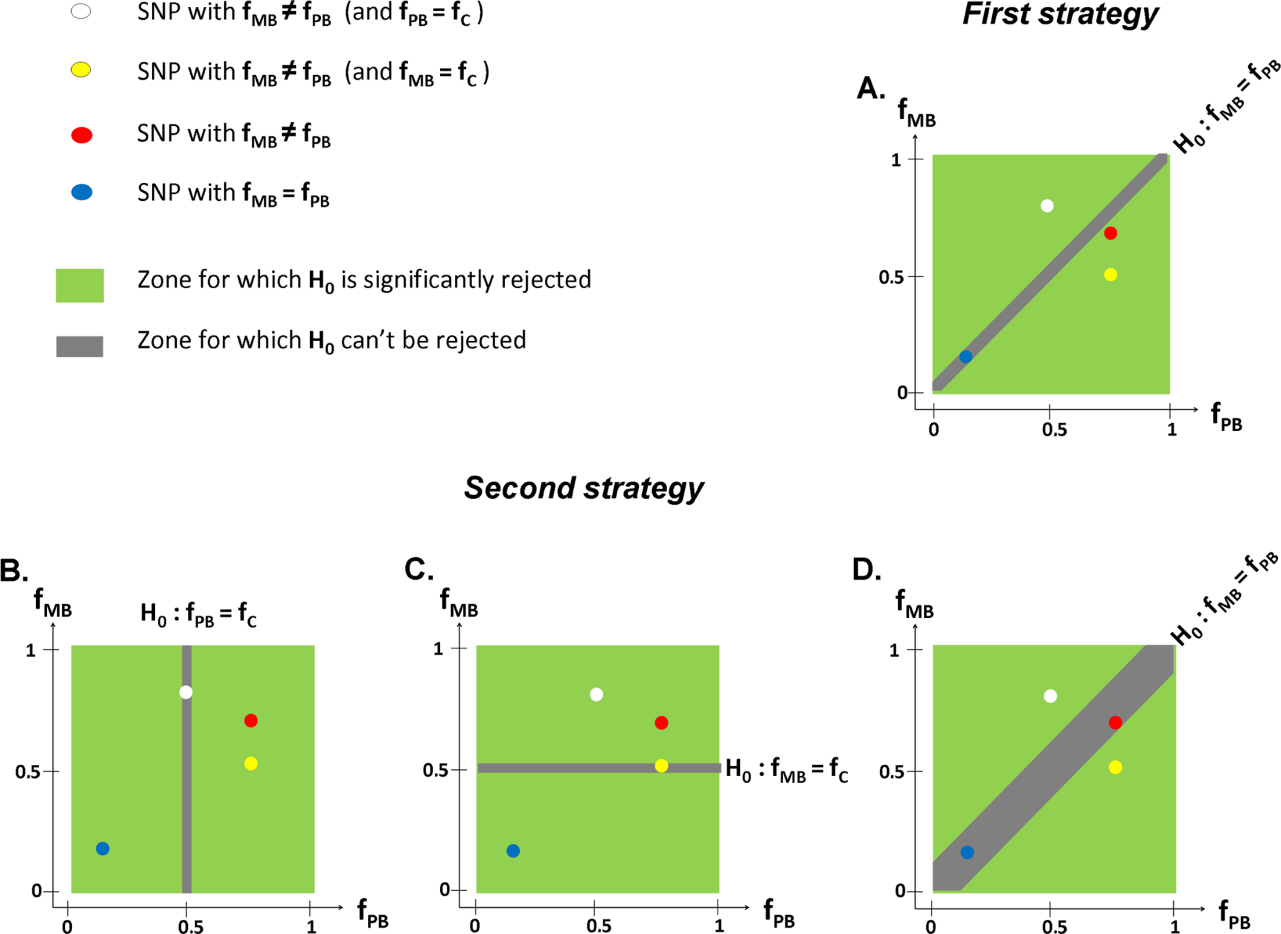
Statistical impact of the hypothesis used to test for an association between a genetic variant and leprosy polarization. In the common case-control study design, a standard test of association between a genetic variant and the phenotype under study is to compare allelic frequencies (f) between the group of cases and the group of controls. Here, we consider a sample including an even number of controls and leprosy cases, equally distributed between PB and MB. In addition, we fixed the frequency of the allele of interest to 0.5 among the controls (f_C_ = 0.5). In each panel, f_PB_ and f_MB_ (x and y axis) are the allele frequencies among PB and MB individuals, respectively. The **first strategy** directly compares MB to PB cases (Panel A). The **second strategy** first tests PB versus controls (Panel B) and MB versus controls (Panel C) before performing a heterogeneity test MB versus PB, possibly after testing leprosy per se versus controls (panel D). The red SNP is a variant for which f_MB_ ≠ f_PB_ and for which the association is significant with the first strategy but not with the second (the red SNP is in the gray area where the null hypothesis H_0_ cannot be rejected). This second strategy is indeed hampered by correcting for multiple testing (testing MB versus PB after testing PB versus controls and MB versus controls) as well as the usual low power of heterogeneity tests.

The optimal strategy to identify variants that specifically differentiate MB from PB patients requires (1) refining the phenotype, (2) balancing the number of patients in each class, and (3) testing the polarization phenotype as a whole. In Fig 4, we give an example in which this optimal strategy can reveal a significantly associated SNP that the classical strategy (testing leprosy per se first and second its subtypes) could not highlight because of a lack of power.

Clearly, it is vital to use specific experimental designs according to the hypotheses to be tested and not to limit future investigations to the simple subanalysis of leprosy per se studies. In particular, comparing MB (or PB) individuals with controls does not allow determining if tagged genes are implicated in the infection, the occurrence of the particular form of leprosy, or even leprosy per se. By contrast, testing MB versus PB individuals is a powerful strategy to identify “specific” polarization genetic determinants. Another part of the study design is the choice of the genetic variants to be tested: it can either be DNA (SNP or any structural variant, such as copy number variant), RNA, or epigenetics (methylation or glycosylation status, for example). Finally, the size of the genotyping effort (candidate gene, candidate region, GWAS) is critical, since the multiple testing will be much less an issue when testing few strong variants in a candidate gene as opposed to several millions of SNPs in a GWAS. In the face of limited resources and relatively small sample sizes, a focus on strong candidates can provide a very substantial increase in power. In the next section, we argue that several powerful strategies able to generate appealing candidate genes have been surprisingly overlooked in the case of leprosy and its subphenotypes.

## Generation of Candidate Genes

### The human track

Because *M*. *leprae* cannot be cultivated in vitro, human studies have emerged as the only source for genetic studies. Candidate genes, are mainly chosen from a list of genes involved in the human immune system: MHC, TLR, and cytokine family genes have been the most popular choices [1,2]. By contrast, “candidate pathways” have never been tested. Definitions of pathways can be derived from the screening of in silico databases such as the Kyoto Encyclopedia of Genes and Genomes (KEGG), Gene Ontology (GO), or Search Tool for the Retrieval of Interacting Genes (STRING). Pathway-based approaches allow extending the search for genetic markers (most typically SNPs) significantly associated with a phenotype from a single gene (~2–10 SNPs) to the pathway level (~100–1,000 SNPs, depending on the size of the pathway). Similarly, pathway-based approaches can be used in the context of GWAS. Such strategies have been used successfully in cancer research [88,89], but have not been reported yet for leprosy.

Large GWAS remain a promising approach for the study of leprosy [90]. However, GWAS rarely have sufficient power to identify genetic variants that are rare at the population level. Such rare variants may potentially have strong biological effects and contribute strongly to the onset of the disease in a given individual. Such variants, although by themselves of minor importance at the population level, can be critical to highlight pathways, as illustrated by the implication of the IFNγ–IL-12/23 pathway in tuberculosis through the detection of private mutations causing susceptibility to mycobacterial diseases in a limited number of individuals. The knowledge of such “Mendelian” or “monogenic” causes of leprosy is currently lacking. Yet, the identification of such cases should be of high priority because of the dearth of relevant in vitro and in vivo experimental models for leprosy.

### The armadillo track

The nine-banded armadillo is a natural host of *M*. *lepra*e that can be used for the study of the in vivo propagation of the bacilli [91–93]. Armadillos are cat-sized mammals found only in the Americas that can live for up to 12 years and have a cool body temperature (32–35°C). The majority of nine-banded armadillos inoculated intravenously with *M*. *leprae* develop a disseminated form of the disease that is similar to multibacillary leprosy in man and, in particular, exhibits severe nerve lesions [94]. A study that included 392 wild armadillos affected by the disease in the United States showed that more than 80% were MB according to Ridley–Jopling (72% LL and 11% BL), regardless of their geographic origin (either from Louisiana, an enzootic region, or from Florida, a nonzoonotic area). Still, 17% were classified as PB. Hence, while armadillo is usually considered as a model for multibacillary leprosy, it can also be used to study polarization. The armadillo genome has been sequenced and annotated [95,96]. Although to date only one armadillo has been fully sequenced, additional sequencing will enable comparison of genetic variants between mammals that develop leprosy (only armadillo and human) and those that do not. Any variants unique to armadillo and humans might offer valuable insight into susceptibility, while genes tagged for comparisons of MB and PB armadillos might provide useful insight into leprosy polarization.

Armadillos also have a unique characteristic that makes them particularly interesting for genetic studies, which is polyembryony (always giving birth to monozygotic quadruplets). This peculiarity can help distinguish between environmental and genetic factors that favor the phenotype. Adams et al. observed that within each set of sibling armadillos experimentally infected with *M*. *leprae* and bred in the same environment, the results of bacteria harvests were similar: this is a strong argument in favor of a major role of host genetics in the ability to fight the bacteria [91].

### The pleiotropic track

In the absence of functional studies specific to leprosy, some genes have been explored in other diseases that share a common genetic background with leprosy. Inflammatory bowel disease (IBD) is a striking example. Genetic studies have revealed common signaling pathways between leprosy and Crohn’s disease (CD) [97,83,2], fostering an ongoing debate about a possible contribution of mycobacteria to CD [98–101]. The analogy can be pursued further by comparing the two main forms of IBD, CD and ulcerative colitis (UC), with the two clinical forms of leprosy. Indeed, PB leprosy and CD preferably involve Th1 signaling, while MB leprosy and UC involve a Th2 response [102]. GWAS on IBD have identified 163 loci associated with IBD: 110 were common to both diseases, 30 specific for CD, and 23 for UC [103]. Among the genes specific for CD, *RIPK2*, *LACC1*, and *NOD2* have been shown to be associated in leprosy studies but not specifically with the PB form (*NOD2* and *RIPK2* with leprosy per se and *LACC1* with MB leprosy). To systematically test our hypothesis that CD and PB leprosy on the one hand and UC and MB leprosy on the other hand share some specific susceptibility genes, it will be necessary to replicate CD- and UC-specific genes in PB and MB subsets, respectively. If correct, this approach will allow scientists working on leprosy to use advances in the genetics of IBD to derive an architecture for the genetic determinants of leprosy.

## Conclusion

Here, we summarized the accumulated evidence for susceptibility genes acting at the polarization level that do not influence the onset of leprosy disease. We highlighted that precise phenotype characterization is crucial for the identification of genes driving polarization. Since there is no conventional animal model for leprosy while armadillo is not widely available, the “reverse genetics” approach through genetic epidemiology remains an attractive strategy to decipher the physiopathology of this neglected tropical disease, of which polarization is a major aspect. Importantly, such progress can only come from a close collaboration between physicians, biologists, epidemiologists, and geneticists. Leprosy, so long a cause of social isolation and stigmatization of those who suffer the disease, will only be eradicated by a joint effort of all components of the scientific community.

**Key Learning Points**

- Age and sex are two independent risk factors for leprosy polarization.
- Males who develop leprosy at an older age display a higher proportion of the multibacillary form as compared to younger affected individuals.
- The most powerful method to identify genes or markers associated with leprosy polarization is to directly compare multibacillary with paucibacillary patients (a design used by a minority of leprosy genetic studies).
- The nine-band armadillo model may prove very helpful in the search for genetic determinants of leprosy and, particularly, its polarization.

**Top Five Papers**

1. Misch EA, Berrington WR, Vary JC Jr, Hawn TR (2010) Leprosy and the human genome. Microbiol Mol Biol Rev MMBR 74: 589–620. doi:10.1128/MMBR.00025-10.
2. Mira MT, Alcais A, Di Pietrantonio T, Thuc NV, Phuong MC, et al. (2003) Segregation of HLA/TNF region is linked to leprosy clinical spectrum in families displaying mixed leprosy subtypes. Genes Immun 4: 67–73.
3. Zhang F-R, Huang W, Chen S-M, Sun L-D, Liu H, et al. (2009) Genomewide association study of leprosy. N Engl J Med 361: 2609–2618. doi:10.1056/NEJMoa0903753.
4. Guerra-Silveira F, Abad-Franch F (2013) Sex bias in infectious disease epidemiology: patterns and processes. PLoS ONE 8: e62390. doi:10.1371/journal.pone.0062390.
5. Kirchheimer WF, Storrs EE (1971) Attempts to establish the armadillo (Dasypus novemcinctus Linn.) as a model for the study of leprosy. I. Report of lepromatoid leprosy in an experimentally infected armadillo. Int J Lepr Mycobact Dis Off Organ Int Lepr Assoc 39: 693–702

## Supporting Information

S1 Appendix Methodology for the collection of leprosy cases in Vietnam. (DOCX)

S1 Table Main types of analyses used in genetic epidemiology. (DOCX)

S1 Fig Proportion of multibacillary patients according to age and sex in a Vietnamese sample. Proportion of multibacillary patients according to age and sex among 1,127 leprosy cases (WHO-82 classification).
(TIF)
