# Supplementary material for: Pauci- and Multibacillary Leprosy: Two Distinct, Genetically Neglected Diseases
Source: PLoS Negl Trop Dis. 2016 May 24;10(5):e0004345. doi: 10.1371/journal.pntd.0004345 (PMC4878860; doi:10.1371/journal.pntd.0004345)
Supplement: S1 Appendix — (DOCX) [file pntd.0004345.s001.docx]

S1 Appendix. Methodology for the collection of leprosy cases in Vietnam

Between January 1990 and December 2009 and in close collaboration with the hospital for Dermato-Venerology of Ho-Chi-Minh City (Vietnam), we recruited a large sample of families (parents and children) with at least one child diagnosed with leprosy. The diagnosis of leprosy patients and their subtype classification according to Ridley and Jopling were based on clinical and histological criteria. In addition, physicians also determined the clinical ‘multibacillary’ or ‘paucibacillary’ status to deliver the appropriate treatment. Lepromin testing was not systematically used in the classification. Bacteriological index (BI) was used when available and diagnosis (of leprosy and its subtype) was systematically established by two independent physicians. Informed consent was obtained from all study participants. The study was approved by institutional review boards and health authorities in Ho Chi Minh City, Vietnam, and The Research Institute of the McGill University Health Centre, Montreal, Canada.

Between 1990 and 2009, we enrolled a total of 2,784 individuals, including 1,182 cases of leprosy. Data was available for the number of skin and nerve lesions, BI, Ridley-Jopling classification as well as the two physician’s classification that in practice determined which multidrug regimen each patient should receive.
