# Supplementary material for: Pauci- and Multibacillary Leprosy: Two Distinct, Genetically Neglected Diseases
Source: PLoS Negl Trop Dis. 2016 May 24;10(5):e0004345. doi: 10.1371/journal.pntd.0004345 (PMC4878860; doi:10.1371/journal.pntd.0004345)
Supplement: S1 Table — (DOCX) [file pntd.0004345.s002.docx]

**S1 Table. Main types of analyses used in genetic epidemiology**

|  | | **Sample** | **Nb of markers** | **Aim** | **Design** | **Limitations** |
| --- | --- | --- | --- | --- | --- | --- |
| ***DNA-***  ***independent studies*** | ***Familial aggregation*** | Families | No  genetic  markers | Highlight an excess in the familial risk for the disease under study | Comparison of the risk of developing the disease among relatives of affected individuals or of healthy subjects | No possible differentiation between genetic and environmental factors (common familial lifestyle) |
|  | ***Twins*** | Mono and dizygotic twins |  | Distinguish the relative contribution of genetic factors from other familial factors that contribute to the familial aggregation | Comparison of the phenotypic concordance rate in monozygotic and dizygotic twins | Rarity of cases  Ascertainment bias of mono and dizygotic twin pairs in old studies |
|  | ***Adoptees*** | Adopted children and their siblings |  |  | Comparison of the correlation between children and their natural or adopted parents | Rarity of cases  Age of adoption |
|  | ***Segregation*** | Families  ≥ 2 generations |  | Determine the origin of familial correlations, in particular investigate the effect of a major gene among all genetic and environmental risk factors | Mathematical modeling of the disease risk according to the effects of a major gene, familial correlations and environmental factors and their interactions | Large samples required  Simple genetic models only, otherwise computationally intensive |
| ***DNA-dependent studies*** | ***Linkage*** | Families  ≥ 2 affected siblings | Candidate-region  (2-10) | Map chromosomal regions on the genome containing one or more gene(s) of susceptibility | Study of the correlation between the phenotypic similarity of two related individuals and their genotypic similarity. In the example of affected sib pairs, we test whether, for a given marker, two affected siblings share more than 50% of alleles inherited from their parents | No possible fine localization of the genes of interest (usual region size is 5-10 megabases in the context of a complex phenotype) |
|  |  |  | Genome-wide  (400–10,000) |  |  |  |
|  | ***Association*** | *Case-control design:*  unmatched cases and controls from a same ethnicity  *Familial design:*  ‘Trio’ = one affected child and biological parents | Candidate-gene  (1-10) | Identify in a particular region a genetic variant responsible for an increased risk of disease or in linkage disequilibrium with the functional genetic variant | *Case-control design:*  Comparison of allele/genotype frequencies between cases and unrelated controls for a given marker  *Familial design:*  Comparison of allele transmission from parents to their affected children  Study of genetic imprinting | *Case-control design:*  Delicate choice of controls  Spurious associations (population stratification & linkage disequilibrium)  *Familial design:*  Genotyping of relatives required  Bias towards younger age |
|  |  |  | GWAS  (500,000-5M) |  |  |  |
|  |  |  | Sequencing  (30M-3G) |  |  |  |
