# Supplementary figures and images for: Pauci- and Multibacillary Leprosy: Two Distinct, Genetically Neglected Diseases
Source: PLoS Negl Trop Dis. 2016 May 24;10(5):e0004345. doi: 10.1371/journal.pntd.0004345 (PMC4878860; doi:10.1371/journal.pntd.0004345)

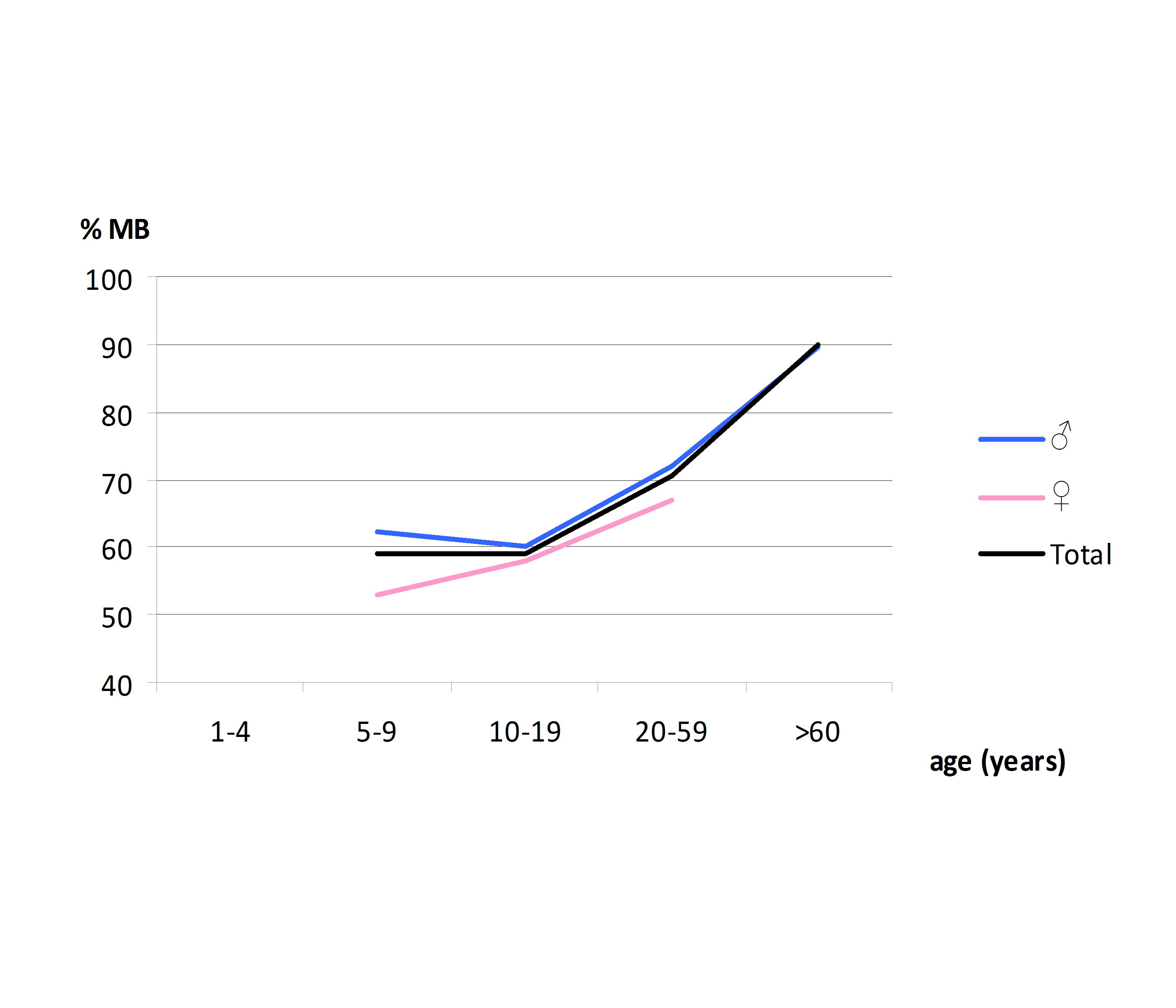

Supplement: S1 Fig — Proportion of multibacillary patients according to age and sex among 1,127 leprosy cases (WHO-82 classification). (TIF) [file pntd.0004345.s003.tif]
